# The Meat Paradox, Omnivore’s Akrasia, and Animal Ethics

**DOI:** 10.3390/ani9121125

**Published:** 2019-12-12

**Authors:** Elisa Aaltola

**Affiliations:** Department of Philosophy, Contemporary History and Political Science, University of Turku, 20014 Turku, Finland; elanaa@utu.fi

**Keywords:** the meat paradox, animal ethics, akrasia, attitudes toward animals

## Abstract

**Simple Summary:**

Psychologists have used the term “meat paradox” to explain why people may emphasize their concern for animal welfare and yet eat meat, the production of which has caused suffering to nonhuman creatures. This paper explores the meat paradox through the philosophical concept “akrasia”. Akrasia refers to a situation, where one believes in a fact or value x, and yet acts against that fact or value. The paper uses the term “omnivore’s akrasia” to denote a state where one believes in the value of animal wellbeing and nonetheless consumes products which have caused animal suffering. The claim of the paper is that understanding of the meat paradox can be significantly broadened with the use of philosophical takes on akrasia, which underline notions such as moral reason and virtue. Another claim is that it is through enhancing one’s moral ability that both the meat paradox and omnivore’s akrasia may be reduced. Specific factors included in such enhancement are introduced and compared with “nudging”. In the conflicting era when the meat industry is rapidly growing on a global scale whilst attitudes toward other animals are becoming increasingly positive, exploring the phenomenon of both eating and caring for animals is of clear societal, political, and moral significance.

**Abstract:**

Western cultures have witnessed an intriguing phenomenon in recent years: People are both more concerned for animal wellbeing and consume more animal products than ever before. This contradiction has been explored in psychology under the term “meat paradox”. However, what has been omitted from the explorations is the age-old philosophical notion of “akrasia”, within which one both knows “the good” and acts against it. The paper seeks to address this omission by comparing psychological research on the meat paradox with philosophy of akrasia. Applying Plato, Aristotle, Descartes, and Spinoza, I investigate the underlying factors of and solutions to what is here called “omnivore’s akrasia”. Whilst contemporary research on the meat paradox focuses on various descriptive cognitive errors (such as cognitive dissonance), philosophy of akrasia has tended to focus more prescriptively on moral reason and virtue. After discussing “nudging” as an implication of the descriptive approach, the paper supports the prescriptive perspective and “the cultivation argument”. The claim is that contemporary research on the contradictions concerning attitudes toward other animals would greatly benefit from paying more attention to the value-laden mental factors underlying moral agency.

## 1. Introduction

The Western world has witnessed a growing surge of interest in the moral status and treatment of nonhuman animals, and this interest has been followed by detailed rational arguments concerning how we, as human beings, ought to relate to other animals. These arguments, provided within animal ethics, have coincided with various kinds of factual evidence, such as knowledge of animal minds, undercover footage from farms, and animal welfare studies, which posit that behind the layers of our cultural depictions of animal industries, lies another, less idyllic reality. In short: We have been offered rational moral arguments and evidence, which support the notion that the way in which nonhuman animals are treated ought to be radically reconsidered. 

Yet, the contemporary era is witnessing an intriguing phenomenon: Individuals, who have been convinced by the moral and factual reasons, are nonetheless often persuaded to maintain the status quo, and to carry on those consumptive habits, which exist in a stark conflict with their values. Indeed, it has been empirically manifested that many omnivores struggle with what in literature is termed “the meat paradox”, within which one both loves and eats animals [1,2]. One can also apply the philosophical paradox, akrasia, to the phenomenon. Within a state of akrasia, one knows x to be true and good, yet acts against x. We can speak of “omnivore’s akrasia” as a state, wherein one believes that nonhuman animals ought not, *prima facie*, to be harmed or killed for secondary reasons, wherein one considers this to imply that the consumption of many, most, or all animal products is morally indefensible, and wherein one yet continues to consume those very products (even when one has access to alternatives). The meat paradox and omnivore’s akrasia appear as similar states, but there is an important difference. The meat paradox does not necessarily imply going against one’s factual knowledge or reason (but rather, for instance, going against one’s empathic intuitions or “love of animals”), whilst omnivore’s akrasia prioritizes the factual or rational component (the akrates goes against his reason). Keeping this difference in mind, the aim of this article is to explore and compare the two phenomena. More specifically, its goal is two-fold: (1) To apply philosophy of akrasia to everyday animal ethics, and (2) to compare the psychological approach to the meat paradox with philosophical texts on akrasia, thereby mapping out the most effective ways to render animal ethics practically applicable. The aim is not to show that the concept of “omnivore’s akrasia” is in any way theoretically superior to the meat paradox, but rather to highlight how it may help to further illuminate the phenomenon of both having moral concern for animals and taking part in practices that cause them harm.

The meat paradox will be discussed via making use of contemporary psychological studies. Omnivore’s akrasia will be investigated via reference to four classic figures in philosophy—Plato, Aristotle, Spinoza, and Descartes. None of these philosophers endorsed strong pro-animal ethics (in most cases, quite the contrary). Thereby, they did not consider akrasia in the context of animal ethics, nor would they have supported the type of conclusions drawn in this paper. Yet, their philosophies on akrasia can be successfully applied to the issue of consuming nonhuman animals by separating the writers’ reflections on akrasia from their wider philosophical apparatus—focus is on “philosophy of akrasia”, not “philosophy of Plato” or “philosophy of Descartes”. 

The paper’s scope can vary from factory-farmed meat to all animal products. Thereby, it is relative to the ethical beliefs of the person in question. If one believes that only factory-farmed meat is problematic and yet consumes such meat, one is acratic, as is the case when one believes that also dairy and egg production, as well as all meat production, is morally wrong but nonetheless carries on consuming dairy, eggs, and meat. Moreover, it ought to be noted that the meat paradox and omnivore’s akrasia do not form a generic moral psychological explanation for the consumption of animal products: There are many of those, who believe they are following emotive intuitions and/or reason in their consumer habits, in which case they are not akratic or “paradoxical” in their actions (as an example of a philosophical effort to justify the killing of animals, see [3]; as an empirical example of common everyday justifications, see [4]). 

Finally, what follows should not be taken to mean that akratic omnivores or those in the grip of the meat paradox are morally “wicked”—indeed, arguably most people (including the author of this paper) are akratic and contradictory in various ways, as we may, for instance, drive cars or fly despite of our knowledge of the severity of climate change. Instead of moral blame, the phenomena deserve the type of attention that may lessen their hold on us (I have explored omnivore’s akrasia previously in [5,6]). 

## 2. The Meat Paradox

Most commonly, the meat paradox is explained via reference to *cognitive dissonance*, within which one holds onto mutually contradictory beliefs or emotions. Thus, belief in the value of animal wellbeing and life may be held at the same time as the belief that one may eat animals, whose wellbeing was poor. Here, eating animals takes place as if in a different conceptual reality from one’s recognition of, say, animal welfare issues [1,7]. Cognitive dissonance often implies *dissociation*, whereby one omits to acknowledge the animal behind the meat—the origin of meat is dissociated from living animals, as “meat” and “animals” become two unrelated categories. Studies manifest that cognitive dissonance and dissociation are powerful sources of the meat paradox, and lessen both empathy toward animals and disgust toward their killing [8,9,10]. They also come with cultural or social dimensions. According to research, particularly dissociation is facilitated by meat marketing, which tends to avoid references to live animals, and within which animal products carry little evidence of their animal origins (such as animal heads, hair, eyes, or blood) [8]. Marketing makes use of the notion that animals are “food”, and this further hides the living animal from view. Indeed, describing animals as “food” diminishes the animals’ perceived capacity for suffering and thereby concern for their welfare [7]. In other words, when one dissociates animals from “meat” or “food”, one begins to have less concern for animal-related moral values. 

*Strategic ignorance* is another common theme behind the meat paradox, and nourishes both cognitive dissonance and dissociation. Here, one undergoes a state of ambiguity or denial by willfully ignoring beliefs that one deems as threatening to one’s choices—puzzlingly, one both recognizes those beliefs and seeks to avoid them. A practical example is blocking out information available on animal minds, suffering or welfare issues, which again facilitates meat-eating. According one study, 27%–28% of individuals use strategic ignorance in regard to meat-eating [11]. Particularly underestimating the cognitive capacities and agency of other animals—the “dementalization” of nonhuman beings—has been proven to be a common feature of strategic ignorance [12,13,14]. Furthermore, studies have suggested that hedonistic enjoyment of meat and the reluctance to change one’s eating habits are two further powerful factors behind the meat paradox [15]. More specifically, underscoring hedonistic values (“meat tastes good”) and custom (“meat is traditional”) are utilized so as to lessen awareness of the paradox [16]. Arguably, also *hedonism* and *habits* can entwine with cognitive dissonance, as one may simultaneously maintain that animals ought to be treated well, and advocate the eating of poorly treated animals on account of the culinary values of, or traditions related to, say, turkey roasts; for the same reason, they can also support dissociation, whereby “the turkey roast” is categorically separated from living turkeys.

The last significant factor highlighted in psychological studies is avoidance of *empathy*. It entwines particularly with dissociation and strategic ignorance, for as soon as one dissociates meat from the living animal or downplays animal mindedness, the creature with whom one could empathize disappears from the scene. In short, if one pays little or no attention to the living, cognitively able, conscious animal, it becomes unlikely one could empathize with pigs or cows. Also, hedonism and habit have been linked to the marginalization of empathy [8,15,16]: if one underscores the taste of a steak or the custom of eating meat, it may become uncomfortable to fully empathize with pigs and cows. Since it can be argued that empathy is a necessary part of a fully developed moral agency also in relation to how we value and treat nonhuman animals [17], diminishing it in the context of making dietary choices can be detrimental from an animal ethics point of view.

One suggestion often brought forward in studies on the meat paradox is that its hold can be diminished by avoiding the previous factors. In other words, one is to lessen the occurrence of, for instance, cognitive dissonance, dissociation, and strategic ignorance. One simple method of doing so is to re-introduce concepts concerning living, minded animals and their poor treatment into the setting of buying animal products or eating them, thereby discouraging the above factors and inviting empathy toward animals. Consequently, the claim is that reminding individuals of the animal origin and the animal cost of their food has the potential to overcome the pull of various forms of disengagement [13]. Indeed, studies suggest that already prompting individuals to consider the meat–animal connection reduces their attachment to meat [18], and that providing images of animals with meat recipes renders meat less inviting [13]. Empirical research also manifests that if individuals are told about the suffering faced by animals in animal industries, they begin to re-evaluate meat: They rate the meat as less tasty, deem it to smell less inviting, and also rate the way it looks as less appealing. Moreover, their willingness to eat meat diminishes, and they state that they would pay less for it [19]. 

Thereby, one obvious solution to the meat paradox is to bring forth the connections between minded animals, suffering, and meat. Importantly, this refocus should not only take place independently from the practices of consuming animals, for doing so may only reaffirm the boundaries between the conceptual reality of “animal ethics” and that of “meat-eating”. In contrast, reminders ought to be brought into the context of also buying animal products and eating them. By repeatedly highlighting animal agency and animal ethical issues within the setting of meat (or dairy and eggs), the separateness of conceptual realities may slowly be eroded, which again lessens the meat paradox. Moreover, since marketing has a tendency to spark and affirm the meat paradox, such counter-reminders need to be sufficiently frequent and widely accessible.

Contemporary psychological discussion on the meat paradox has much merit, particularly due to its interest in the complexities of animal-related decision-making. However, it tends to omit that a phenomena related to it, akrasia, has been explored in Western philosophy for 2500 years. Considering the breadth of philosophical analysis on akrasia, this is a significant omission. In order to better understand the discrepancies between animal-related values and actions, and thereby to map out ways for a more animal ethically sound future, also the philosophical analysis of akrasia deserves focus. 

## 3. Plato: The Art of Measurement and Self-Control

Socrates was famous for his claim that immorality is ignorance. Once we have adequate knowledge, we will not fail to do what is good. This assumption has colored much of political advocacy, including efforts to better the moral standing of nonhuman animals. Hence, providing people with more knowledge concerning animal minds or suffering is often presumed to convince them to change their consumer actions. Yet, something appears to be amiss here. People frequently make choices against their better judgment. For instance, at the same time as many in Sweden state their willingness to reduce their meat-eating, consumption of meat has peaked [20]. 

Plato, in whose dialogues Socrates offered his views, acknowledged that one may misjudge the moral nature of a given situation, and thereby do wrong even when one thinks one’s actions are good. In *Protagoras*, Plato suggests that “good” and “bad” are in practice often based on pleasure and pain—that, which leads to pleasure, we tend to deem “good”, and that, which leads to pain, is often deemed “bad”. However, one may mis-measure the affective consequences of an action, and thus for instance prioritize a relatively small short-term pleasure instead of significant, long-term pain—the Platonic cause for why people eat or drink too much in order to satisfy their immediate desires and pleasures, whilst more significant, long-term consequences filled with pain are ignored [21,22]. Indeed, Plato argues that precisely such mismeasurements concerning affective consequences lead people astray, as they spark appearances, which mask “bad” as “good”, and vice versa. It is this mechanism of *mismeasurement* and *appearance*, which spurs one’s failure to follow what is true and good. The term “akrasia” applies here, when one has an inclining towards “the good”, but momentarily latches onto false appearances. On these lines, Plato claims that people are frequently confused about what is good and how to act: “The power of appearance often makes us wander around in a state of confusion, often changing our minds about the same things and regretting our actions and choices” (Protagoras 356d) [21]. 

According to Plato, the solution to akrasia is simple: We are to cultivate our capacity to measure and judge our pleasures and pains appropriately. This skill he likens to doing arithmetic, and it requires the use of reason: “The art of measurement […] would make the appearances lose their power by showing us the truth” (Protagoras 356d). Another solution to akrasia, suggested by Plato, is *self-control*, whereby one is to govern one’s desires so that they can no longer misguide. In *Gorgias*, Plato (again, via Socrates) argues that self-control is nothing short of necessary for happy and moral lives, for which reason “Each of us must flee away from lack of discipline as quickly as his feet will carry him” (Gorgias 507b). A mark of self-control is one’s ability to “avoid and pursue what he should” (Ibid.) [21], and thereby to do good, which renders it a gateway away from akrasia.

Plato’s claims are relevant to also omnivore’s akrasia. Arguably, such akrasia can originate from one’s mismeasurement between pleasure and pain, with one important twist: Here, the pleasures and pains of the animal are ignored. Thereby, the error is to calculate the goodness of an action by overlooking the affective consequences it has for the animal, as one prioritizes the short-lived human pleasure of eating factory-farmed steak over the severe, long-term suffering underwent by the animal. Perhaps many akratic omnivores are misled by the afore-mentioned appearances, as the failure to recognize animal pains causes them to mis-measure the situation. This, again, may feed the sort of confusion detailed by Plato. On the one hand, the individual values animal wellbeing and lives highly, and on the other, he prioritizes his own secondary enjoyment, thereby vacillating in a state of ambiguity and unresolved tension, one minute believing x, and the next minute believing it just to act against x. The solution would be to cultivate one’s ability to measure appropriately all the relevant pleasures and pains, whereby the pleasures of meat-eating (or consumption of dairy and eggs) would be compared with the woes of animal industries. Thus, omnivore’s akrasia could be lessened by learning the art of measurement, and by including in one’s measurements all the salient factors, also beyond the species borders.

Moreover, I propose that the akratic omnivore would profit also from cultivating self-control so as to follow “the good”. Most of us know the practical dilemmas of believing x, and yet wanting to do something that violates x, whether this is in relation to overindulgence on food or drink, protracted idleness or flying to holiday destinations in the era of climate change—moreover, most of us are probably capable of recognizing the lack of self-discipline underlying this phenomenon. It appears that such lack can spur also omnivore’s akrasia, whereby one rates the moral importance of animal wellbeing highly, but does not have the required self-regulation to act in ways that support that wellbeing. The solution would be to strengthen one’s character toward *enkrateia* or self-control, the trait or virtue that many philosophers from the Antiquity depicted as one key pillar of morality. Here, the akratic omnivore would simply resist the temptation to consume products, which have caused suffering and death to others (provided that there are alternatives). Of course, such reminders of self-control may not be appealing in the contemporary, consumeristic era, which is marked by the logic of marketing that precisely rests on one’s lack of discipline. Yet, precisely because of this, the contemporary akrates would benefit from appreciation of self-control, as it could—following Plato—ensure happier, fuller, and morally more illuminated lives. In the context of animal ethics, this is particularly vital, since (as implied above) marketing significantly increases the consumption of animal products. 

But how to cultivate self-control? Self-control requires self-knowledge, whereby one is capable of mapping out one’s underlying motivations. Socrates urged us to examine our lives, and “know thyself” was a proclamation frequently emphasized in Antiquity (Apologia 38a5–6). By becoming more familiar with our motivations, we can learn to govern them more comprehensively. Self-knowledge allows the akratic omnivore to re-evaluate his pleasures, wants, emotions and other motivations, so as to make more measured judgments in line with information concerning animal minds, needs and treatment, and ultimately so as to have more control over his own moral character and actions. Here, the question of what type of beings we are and wish to be in our dealings with other animals entwines with self-control and the avoidance of akrasia—a question most timely in the era of animal industries, mass extinction, and climate change.

## 4. Aristotle: The Virtue of Temperance

The descriptions of akrasia available in Aristotle’s ethics are varied and, partly due to this variance, capable of offering a heterogenic insight also into omnivore’s akrasia. According to the most discussed Aristotelian interpretation, akrasia refers to a lack of the above-mentioned self-control (indeed, it literally means “lack of mastery”) [23]. It emerges as a form of *malakia* or feebleness of character, and thereby stands as the opposite to *enkrateia* or *karteria*, which denote moral control, mastery and endurance, the ability to follow through one’s conviction even when various factors form formidable obstacles. Indeed, according to Aristotle, akrasia is “a type of softness”, born out of the incapability of the akratic person to resist temptations that can mislead toward wrongful actions (NE 7.7.1150b1-3) [24]. As a result, the akrates is left to exist “in such a state as to be defeated even by those [obstacles] which most people master” (NE 7.7.1150a11-13). The “obstacles” that the akrates is unable to overcome are formed of pleasures and passions. The akrates knows what he ought to do, but his desire for pleasure, or his emotions, push toward the other direction: “But there is a sort of man [...] whom passion masters so that he does not act according to the right rule, but does not master to the extent of making him ready to believe that he ought to pursue such pleasures without reserve; this is the incontinent man” (NE 7.8.1151a20-27). Aristotle has two metaphoric examples for such resolution-lacking “incontinence”. First, it is as if the incontinent person is drunk, intoxicated by desires or habits that push him to commit acts he knows to be wrong (NE 7.8.1151a4-5). Second, akrasia is likened to an illness such as epilepsy, with temporary, disabilitating seizures (NE 7.7.1150b12-14).

When applied to akratic omnivores, the above suggests what was already discussed in relation to Plato’s views: They may be led by poorly judged desires (and, as Aristotle adds, emotions). Applied to the present context, the desire for a meaty cheeseburger, the desire to fit in a meat-eating society, and emotions such as happiness over a shared, traditional meal, may spur one to ignore animal-related values and ethics. Perhaps the omnivorous akrates is even as if “intoxicated” by his taste buds used to particular flavors, or as if “ill” with the incapacity to resist the temptations culturally linked to meat-eating. This disables the akrates from following moral judgment, and renders him into the prototypical nightmare of the animal advocate: an individual, who heartily understands that animals ought to be treated with respect, but who nonetheless stubbornly acts directly against that understanding. 

Another commonality between Aristotle and Plato is the belief in self-control, and applying this to animal ethics, the akratic omnivore fails to govern his desires and emotions, and to steadfastly abide by his animal ethics. Yet, whilst Aristotle paints a rather unforgiving picture of the akrates, a more sympathetic approach is warranted here. Humans as highly social creatures tend to follow socially widespread habits, and thereby it is little wonder if one’s moral self-control may waver when it comes to habits as ingrained to most human societies as meat-eating. Aristotle held an optimistic view of society and its ability to teach us ethics, and for him the act of following widely accepted customs was an important part of internalizing virtues. However, a more realistic take on society notes its conflicting moral messages and the often morally ambivalent or problematic nature of dominant habits. In short, not all widely accepted habits are morally sound, and society’s impact may even render us akratic. Indeed, following Amelie Rorty’s adaptations of Aristotle, it can be argued that akrasia ought to be approached also and even primarily as a social phenomenon, sparked by the confusing messages we get from the social institutions surrounding us [25]. This would mean that also omnivore’s akrasia stems largely from social contradictions (we are told to both love animal wellbeing and lives, and eat dead animals, who suffered), which underlines the power of social causes behind one’s desires and lack of moral resolution. This highlights something pivotal: One solution to omnivore’s akrasia lies, not only in developing self-control so as to govern misleading desires and emotions, but also in reflection on and reform of social beliefs and habits related to nonhuman animals. In particular, attention should be focused on the contradictions in how actions toward nonhuman beings are defined and evaluated within different institutions and settings (why love or empathy is the dominant norm in one instance, and killing and eating the status quo in another) [5]. 

A further exploration of Aristotle’s conception of akrasia is in order. As Devin Henry has emphasized, for Aristotle, pleasures are divided into *alien* and *proper pleasures* [26]. Whereas alien pleasures are base and antagonistic toward virtue (for instance, they tend to come in bodily excesses), proper pleasures derive from virtuous activity; they enable us to flourish and fulfil our telos. Aristotle specifies: “Activities are made precise and more enduring and better by their proper pleasure, and injured by alien pleasures” (NE 10.5.1175b14-17). Importantly, proper pleasures are accompanied by *temperance*, the ability to detach from alien pleasures. Following the logic of virtue, in order for us to resist the cruder human inclinations, they need to be equipoised by their opposites, i.e., the higher forms of pleasure, and we are to choose the half-way between the extremes. Here, being temperate in itself becomes a form of pleasure, a source of enjoyment, as we begin to rejoice over our capacity to moderate our actions and stay clear from corrupt inclinations (greed, hedonistic gluttony, and so forth). In fact, it is lack of temperance, which genuine akrasia, according to Henry, is ultimately founded upon: A person undergoing it cannot resist the pull of base appetites, the lure of alien pleasures, for there are no counterbalances for them, no capacity for restraint [26]. 

Following suit, temperance emerges as one cure for akrasia. First, we are to entertain proper pleasures, to find the sort of weight in them that balances our internal scales, and second, we are to rejoice in our ability to moderate our action. Thereby, it is the capacity to navigate internal conflicts, the various pulls and tugs toward opposite directions, and the capacity to draw joy from such navigation, which surface as solutions to akrasia [26]. This, again, requires moral training. Aristotle explicates: “To enjoy the things we ought and to hate the things we ought has the greatest bearing on moral excellence” (NE 1172a22-24). Again, Aristotle echoes Plato to some extent. However, where Plato urges us to measure pleasures accurately and compare them against each other, Aristotle suggests we ought to learn to enjoy temperance and the midway between opposing motivations. 

Perhaps, then, the akratic omnivore would benefit from learning (1) proper pleasures attached to the human–animal relationship, and (2) the joys of temperance. Omnivore’s akrasia would, within this interpretation, consist of alien pleasures, intertwined for instance with habitual hedonistic pulls (“I want dairy ice-cream, even if it comes from maltreated cows”). As things stand, these pleasures often lack the counterweight required for temperance, and thus the omnivorous akrates slides away from balanced decisions into actions driven wholly by, for instance, the wish for a particular culinary experience. In order to nourish proper pleasures and temperance, the akrates would have to step back from those pleasures that cause animals harm, and instead consider how else one might enjoy the nonhuman realm. The proper pleasures could include, following Aristotle’s lead, the virtues of friendship, justice, and practical wisdom. One could deliberate on the (admittedly non-Aristotelian) notion that minded non-human creatures can belong to the sphere of friendship, that they ought to be met via notions of justice, and that one ought to practice practical wisdom and consideration as it comes to their flourishing, telos and wellbeing. Alternatively, the proper pleasures could consist of more modern virtues. Here, for instance varieties of empathy, the desire to understand other animals better in all their similarity, peculiarity and difference, and ultimately the desire to enhance one’s moral regard for those animals, would form the proper pleasures that spark temperance. 

In both instances, proper pleasures would manifest a higher form of art, considerably more worthy of pursuing than pleasures that unnecessarily harm animals, and offer the type of a counterweight required for temperance to emerge. As a consequence, one would delight at witnessing alive and well animals manifesting their telos out in the wilderness or in the domesticated/feral setting, and draw gladness from being able to perceive them as cognitively capable subjects and creatures worthy of consideration, justice, empathy and even friendship. Ultimately, “animal-directed temperance” itself, one’s capacity to approach nonhuman animals with reflection and virtue, could spark gladness. In fact, a psychological, moral reward (enjoyment of proper pleasures and temperance) may be needed to support the otherwise rather uninviting notion of self-control, and may stand as necessary in eradicating omnivore’s akrasia. The omnivorous akrates is freed from his paradoxical state when he learns that manifesting animal ethics on the level of action is, with its capacity to make us flourish as just, empathic, morally considerate beings, a matter of pleasure, virtue and happiness. 

## 5. Descartes and Spinoza: Generosity and Joy

In order to map out what (early) modern philosophy had to say on akrasia, I will briefly discuss René Descartes and Baruch Spinoza. Descartes examined akrasia particularly in his *Passions of the Soul* [27]. Like Plato and Aristotle, he suggested that desires may have a role to play; he also claimed that habits and mental shortcomings may drift one’s attention away from what is good. Most crucially, however, whereas Plato and Aristotle offered self-control as one method out of akrasia, Descartes highlighted one’s rational *will* as an alleviation to akratic confusions, whereby we rationally and actively seek to live in accordance with our values. Such will emerges as a solution to akrasia simply, because one *wants* to do what is rationally good. Significantly, in the moral context, the will is related to *generosity*, the readiness to priorities also the wellbeing of others, and to support it by even self-sacrifice. It is particularly in a state of generosity that we strive to follow in our actions that which is also rationally just [28]. It is a combination of virtue, emotion and self-control, which prompts us to govern desires and attention so that they follow the rational, morally reflected will also, when that will dictate that we give from ourselves to others [29]. Descartes clarifies: “[t]hose who are Generous in this way are naturally inclined to do great things... and because they esteem nothing more highly than doing good to other men and for this reason scorning their own interest, they are always perfectly courteous, affable, and of service to everyone” (p. 156) [27]. 

Therefore, akrasia may originate from mental shortcomings (such as misplacement of attention) and lack of a generous will. The akrates fails to focus on “the good” adequately, and may have a diminished capacity to actively *will* the good of also others. Descartes’ suggestions find support in contemporary empirical studies on akrasia. States related to lack of attention and the incapacity to follow one’s will, such as impulsivity, ambivalence about what to do and apathy, can feed akrasia [30]. Moreover, studies on the role of executive functioning, an ability often linked to both attention and the philosophical notion of “the will” (here to be separated from “free will”), point out its importance for one’s ability to make and follow moral judgments [31] and for “moral maturity” [32]. Executive control includes a variety of traits crucial for moral agency, such as attentional ability, planning, inhibition, information processing, and goal setting [32]. In sum, executive control governs automated conceptualizations and responses, directs attention and motivates goal-orientated action [33], which in turn suggests that it involves or even to some extent correlates with, not only attention and the “will” underlined by Descartes, but also the type of self-control emphasized by Plato and Aristotle. Current research on executive control lends support to Descartes’ claim, according to which the akrates would benefit from cultivating focus on what is moral, and from cultivating also one’s will to follow what is rational and good. In fact, whilst the term “will” has disappeared from contemporary sciences, as the late 19th century psychologists began to substitute it with reference to more specific mental traits (One argument is that forsaking “the will” has been a trend, which may lack justification. Thus, perhaps we ought to bring “the will” back [34].), its most current replacement appears to be “executive function”. Whichever term is used, the importance of focusing and reflecting on, and practically acting so as to follow what is good, is surely still relevant. Both terms accentuate the role of *motivation*—our moral actions require the conscious impetus to put our values into practice. The vital and rather novel reminder that Descartes brings to the contemporary setting is the nature and role of generosity: the active wanting of the wellbeing of also others. 

Applying the above to omnivore’s akrasia, it may be that concern for the wellbeing of other animals remains theoretical—one has a belief that they ought *prima facie* not to be harmed, but the will to follow this in practice remains insufficient. Shortcomings such as impulsive choices on the supermarket aisle, ambiguity over which value to follow, and moral apathy (accompanied for instance by the belief that one individual’s actions count to nothing) can spur omnivore’s akrasia. Following this line of thinking, cultivating attention on, and an active will to do, what is morally reasonable also in regard to nonhuman animals would act as a remedy to the shortcomings, whereby—in contemporary terms—one’s executive functioning and motivation in the context of animal ethics would strengthen. What would surface as paramount is rational focus on why and how one does and should treat animals, accompanied by an active will to follow that focus. 

This, again, could entwine with what I term “cross-species generosity”, i.e., the also emotively felt willingness to give from oneself in order to benefit the flourishing and lives of other animals. Arguably, generosity is often lacking in our dealings with nonhuman creatures. Following the anthropocentric, culturally perpetuated ethos, many assume that human interests always have priority over those of other animals to an extend that secondary human interests trump primary animal interests. As a consequence, other animals are often viewed via the lens of use-value—their ontological status is to exist for human beings (perhaps ironically, such a stance was favored also by Aristotle and Descartes). Here, animals are to give themselves to us, whilst the notion of human beings offering something significant of themselves to animals in actual practice remains rare. The meat paradox and omnivore’s akrasia underline this lack, as often even individuals, who profess to morally love or care for animals, fail to follow this ethic on an applied, practical level in their everyday consumer choices. One possibility is that cultivating generosity toward other animals could even radicalize the human–animal-relationship and everyday animal ethics for one simple reason: Doing so would urge one to recognize that one is to pay serious, practical heed to and even prioritize given animal interests. That is, the notion of “giving of oneself to other animals” would turn the tables on anthropocentric tenets by placing extensive emphasis on animal flourishing and by calling for human beings to be of utility to also pigs, hens, pikes or elks.

Also Baruch Spinoza sought to understand akrasia. He placed emphasis on external influences, such as poorly reflected *opinions*, and their ability to stir *misleading emotions*, such as lust or greed, which again are based on our overt proximity to things. Akrasia is a state of “bondage”, wherein one perceives things without adequate distance and gets engulfed in for instance biased opinions and ensuing emotions, with the result that one’s actions become misguided. Spinoza argues: “Man’s lack of power to moderate and restrain the affects I call bondage”, whereby “though he sees the better for himself, he is still forced to follow the worse” (Ethics IV, pref.) [35]. Indeed, often “We are driven about in many ways by external causes and… like waves on the sea, driven by contrary winds, we toss about, not knowing our outcome or fate” (Ethics III, p59s). In order to avoid akrasia, the task is to follow adequate ideas—the type that reflect nature or existence. These can be found through the use of reason, uncolored by external doxab [36]. The highest form of such reason is grounded on intuitive knowledge *Sub Specie Aeternitatis*, whereby one perceives reality under the category of eternity, thereby gaining “adequate knowledge of the essence of things” (Ethics II, p40s2). Here, one takes distance from the proximity of things, and seeks to observe beings, things and events more holistically, by paying attention to how nature manifests itself through and in them. The consequence of this is joy, which again entwines with vitality or “conatus” and thus by preserving life points toward (or constitutes our conception of) what is “good” (Ethics II p49; III p11). Together reason and joy energize also the will to do good deeds, as they bring us toward “greater perfection” (Ethics III p59s). Through this project, one is released from the bondage of poorly reflected opinions and emotions, and becomes capable of acting in accordance to rational, joyful morality—accordingly, akrasia disappears.

Applying Spinoza’s views to omnivore’s akrasia, perhaps the akrates is following culturally circulated, factually inaccurate and poorly reasoned opinions and beliefs, which support the perpetual marginalization of animal mindedness and interests. Here, one may in theory believe that animals deserve moral focus, but in practice be persuaded by cultural doxa, which position animal consumption as the norm. This, again, may spark or accentuate emotions or other affective dispositions such as greed or lust for meat, which place one under a temporary “meat bondage”. In such instances, the popular opinions and emotions tangled with them may become habits, which have a powerful take on one’s consumer choices, and which mislead away from the moral values otherwise attached to nonhuman creatures. Freely interpreting Spinoza further, one possible answer to omnivore’s akrasia would be the critical evaluation of external pressures and opinions, together with the refinement of reasoned clarity and morally productive emotions. Instead of merely following public opinion, one would be urged to reflect on animal ethics, and to afford space to those emotions, which echo and support such reflection.

Most importantly, perhaps the akrates would find relief from knowledge *Sub Specie Aeternitatis*, thus taking a step away from the proximity of immediate doxa and emotions, and seeking to perceive the animal world holistically as a larger landscape, in which each animal manifests the existence and attributes of nature. In so doing, he could, first of all, locate his own animality, and thereby acknowledge himself within the animal world. Second, he could recognize the situatedness of all animals (himself included) in the broader environmental realm, as creatures displaying the intricacy of the heterogenic whole of nature. Such a holistic standpoint is antidotal to the anthropocentric ethos, which prioritizes *Homo sapiens* and divorces human beings from animal and environmental connections. By observing the ontological relations between species as if from under the category of eternity, one has promise of gaining a broader, more relational understanding of also pigs, hens, and cows. Such an understanding would be capable of noting animals’ complex evolutionary pasts that give rise to equally complex needs and cognitive traits in the presence, and would also remind us of the core resemblances and interconnections between human and nonhuman animals. (Although holistic and individualistic perspectives are often separated in philosophy, giving rise to a drift between environmental and animal ethics [37,38], this could open one door into marrying the two, worthy of closer exploration).

When such an understanding interweaves with reason, joy and conatus, it may spur active commitment to one’s moral values and the willingness to follow them in practice also when making dietary choices. Again, motivation becomes central: gaining more adequate ideas of other animals, and feeling joy in the process, increases vitality also in animal ethical actions, and “the good” concerning nonhuman beings can become apparent. Of course, it is possible one’s knowledge *Sub Specie Aeternitatis* would not lead to perceiving inherent value in other animals—however, the argument here is that *when* it would lead to such a perception, it could offer the kind of potency, motivation and positivity required for one to follow one’s values in everyday life. Thereby, letting go of animal products that harm animal flourishing and lives would no longer be a demanding sacrifice, but rather a positive, energetic action colored by a holistic, joyful commitment to the flourishing of the animal world and its beings. 

## 6. Nudging and the Cultivation Argument

To summaries, the meat paradox refers to a state, where one cares for and yet harms other animals. Research on it posits that it is caused and maintained particularly by six factors: Cognitive dissonance, dissociation, strategic ignorance, hedonism, custom, and marginalization of empathy. The solution brought forward by scholars is empathy and the re-introduction of the living animal into decisions concerning animal consumption, which again entails highlighting animal mindedness and welfare concerns.

The philosophers discussed here offer four different explanations and solutions to akrasia. First, akrasia is born out of misevaluation or mismeasurement of one’s desires and their affective outcomes, and second, it may stem from following unvirtuous or unethical pleasures. Third, akrasia can be the consequence of weakness of will or resolution. Fourth, akrasia can be caused by external opinions and misleading emotions fed by them. In all these instances, one may know what is “good”, but in practice this knowledge gets muddled and ambiguous, whereby one fails to act according to it. The solutions to omnivore’s akrasia that can—with substantial interpretation—be drawn from the above philosophers are as follows. First, one is to learn both the art of measurement and the virtue of temperance, whereby we pay heed to also animal pains and “proper” human pleasures such as animal ethical virtues. Second, one is to practice self-control and the virtue of strength of character. Here, developing good will and generosity may be of help, as one becomes more capable of actively supporting (rather than passively ignoring) the wellbeing of others. Third, reasoned, moral reflection is required. Fourth, knowledge *Sub Specie Aeternitatis* and joy intertwined with it hold promise of rendering animal ethical concerns more inviting also in practice, thereby offering the incentive or motivation to follow “the animal good”.

What the contemporary psychological model on the meat paradox and classic philosophical explanations of akrasia have in common is the suggestion that ethically ill-considered desires, pleasures, habits and opinions can cause the failure to live according to one’s values. Yet, there are also obvious differences between explanations of the meat paradox and akrasia. I will first map out what contemporary psychology can teach philosophy, and second, how philosophical texts on akrasia may help psychological investigations on the meat paradox.

First, the psychological concepts of “cognitive dissonance”, “dissociation”, and “strategic ignorance” offer new tools, with which to make sense of also akrasia. Instead of one simply being in the grips of desire or opinion, one may undergo cognitive states of contradiction, which in themselves trigger akrasia. Thereby, perhaps akrasia is not always caused by an additional factor x (desire, opinion) influencing decision-making, but rather it may be *inbuilt* into various mental conditions, thereby being a phenomenon concerning the structure rather than the content of our mindedness. Second, the philosophers discussed here fail to recognize the role played by empathy, the absence of which appears to be a powerful cause of akrasia. When marginalizing empathy, one may rationally recognize a given value, yet fail to follow it simply, because the relevant emotive incentive is lacking. Arguably, particularly “affective empathy”, with its ability to invite us to resonate with the experiences and viewpoints of others, is morally vital [17], and may often be the encouragement that pushes us to act in a morally sound manner toward minded (human and non-human) individuals. Following suit, philosophical discussion on akrasia would greatly benefit from paying far more focus on empathy and related other-directed emotions. 

But what could contemporary psychological research on the meat paradox benefit from the philosophy of akrasia? First, the role of misevaluating or mis-measuring desires is of significance. Here, references to hedonism do not suffice, for what deserves focus is also the manner in which individuals miscalculate the weight of given desires and pleasures, and thereby follow what for Plato are misleading “appearances”. Second, the classic notion of volition or will deserves more focus also in explorations of the meat paradox. In modern terms, perhaps it is often one’s failures in executive functioning, whether this is due to impulsivity or lack of attention, which leads to consumer choices that go against one’s ethics. Indeed, since the relation between morality and executive functioning has been established empirically, it is surprising that research into the latter’s links to the meat paradox is largely non-existent. Thirdly (and relatedly), the classic notion of conatus or vitality is often absent from contemporary discussions, and merits more attention. If impulsivity erodes “the will” or executive functioning, and thereby leads to poor choices, so does its twin procrastination, whereby one has no urge or impetus to act. In regard to the meat paradox, would it not thereby make sense to invite energizing and motivating emotions, such as joy, to the scene? Fourth, research on the meat paradox could benefit from taking lack of virtue or moral reflection into account. The disinclination to follow one’s values may not stem from only cognitive failures, but can also entwine with failures in our normative engagement with the world. Here, the classic notions of moral reasoning and virtue surface as important: Surely the person undergoing the meat paradox would benefit from being urged to also rationally reflect on his actions toward other animals, and to consider the cultivation of “animal-related virtue”, such as empathy, temperance, generosity, and trans-species justice. 

The latter leads us to the primary difference between contemporary psychological approaches to the meat paradox, and philosophical takes on (omnivore’s) akrasia. Whilst the former focuses mainly on *descriptive* faculties or states, the latter emphasizes also *prescriptive* faculties and states. Whereas scholars studying the meat paradox speak mostly of value-neutral conditions such as cognitive dissonance, thereby explaining why virtuous individuals act against their virtue with reference to non-moral psychological states, philosophers also discuss moral reasoning and various virtues *per se*. There is cause to bring the latter back into contemporary discussion. When analyzing why one errs in following one’s moral values, also the prescriptive moral-psychological mechanisms underlying those errors (for instance, deficiencies in the virtues of temperance and generosity) deserve comprehensive focus—something that studies on the meat paradox have largely omitted. This omission may find roots in the differences between contemporary cognitive terminology and the terminology of philosophy. For the former, concepts such as “virtue”, “temperance”, or “good will” can sound hopelessly archaic, whilst more value-neutral concepts with scientific connotations are favored. Yet, I suggest that overlooking the significance of the older, moral notions is a mistake, and reflects a tendency to steer away from the normative into purely factual considerations that lose sight of the significance of our moral agency and fiber. Indeed, in explorations into why Westerners both love and eat more animals than before, emphasis ought to be placed also on the particulars of our moral constitution. Both the meat paradox and omnivore’s akrasia stem partly from failures in our moral psychological capacities and virtues—and if this is the case, it is those capacities and virtues, which need to be strengthened.

To highlight the importance of investigating moral abilities, an example is in order. As suggested earlier in the context of Descartes, impulsivity and apathy have been argued to play a role in akrasia. According to contemporary research, impulsivity and procrastination (or apathy) had possible evolutionary benefits in the early development of our species (for a person fulfilling his immediate needs, it may have been useful to be impulsive when finding food or escaping dangers, and to take it easy the rest of the time). However, in the modern world, such traits are usually harmful for both oneself and others, for they damage our long-term interests [39,40]. In fact, what links these two otherwise opposite traits is diminished ability for long-term goal-orientation and self-regulation. Now, I suggest that goal-orientation must and should include also moral concerns on a meta-level: the cultivation of our moral agency or moral maturity ought to be one of the primary long-term goals of our species. If we are to learn how to live in a manner that supports life on this planet and the wellbeing of other species and their individuals, and if we are to also learn how to keep *Homo sapiens* alive, strengthening our moral fortitude is necessary. Significantly, the long-term goal of cultivating moral agency and most of our other long-term goals (such as survival) are interrelated—something that is far too frequently forgotten. Current psychological research on “life history theory” has pointed out that for individuals living in unpredictable environments, short-term rewards, impulsivity and “fast living” are more optimal, whereas for individuals in more harmonious environments, long-term goals and measured life-plans are given [33]. I argue that the relation may run also the other way, whereby emphasis on long-term goals can feed more harmonious existence; moreover, I would add that cultivation of moral agency ensures such an emphasis. Therefore, with moral ability and maturity, we may be better able to stay tuned to long-term goals and reach calmer living conditions.

The implication is that one ought not only fight impulsivity and procrastination (or indeed cognitive dissociation or dissonance), but also develop one of their antidotes—moral ability. The central questions become: What sorts of beings do we wish to be in relation to members of other species? How might we improve our moral ability in order to lead good lives whilst also supporting the good lives of other animals? In order to put our animal ethics into practice, and thereby to avoid the meat paradox and omnivore’s akrasia, it is these questions of moral learning, which become primary. I call this “the cultivation argument”: When addressing contradictions in our treatment of other animals, emphasis should be on rendering our moral psychological constitution better able to pay practical heed to also individuals of other species. It is this that philosophy on akrasia highlights: The need, not only to address descriptive mental states that cause conflicts between actions and values, but to also cultivate moral ability. The relevance of the latter project becomes more apparent when considering a recent trend in affecting dietary choices—nudging.

Daniel Kahneman has popularized the claim that human behavior is governed by two distinct systems. “System 1” is automatic and unconscious, whilst “system 2” includes conscious deliberation [41,42]. Although this distinction may sound simplistic, it holds much explanatory power. There is growing empirical evidence that the rational behavior model (or “the rational choice theory”), according to which consumers make decisions based primarily on information, is mistaken, as people routinely fail to make rationally deliberated choices [43,44]. When it comes to consumer decisions, it is predominantly system 1 rather than system 2, which is in charge [45,46]. Thereby, offering individuals more information on issues such as climate change or diet has been found to have little impact on their everyday choices [47]. In fact, the majority of consumer decisions go against the rational beliefs held by the consumers. Factors such as cognitive biases, cost aversion, favoring short-term goals, habits, and mental shortcuts play a large, unconscious role in our everyday dietary choices [41,43,47,48,49]. All of this lends support to the prevalence of akrasia in decisions concerning what to eat, and underlines the manner in which also omnivore’s akrasia may be a widespread phenomenon. 

Since the causes behind acratic behavior are largely unconscious, it has been argued that its eradication requires the help of structural and environmental interventions able to affect us on the unconscious level [50]. This has led to the notion of “nudging”, which refers to altering consumer behavior on a level beyond awareness without restricting people’s options. Nudges consists of using unconscious biases in favor of rather than against important values, such as health or sustainability—the system 1 shortcuts are utilized via “choice architecture” so as to invite beneficial decisions [49]. Examples include change of defaults (making moral choices the “default option”), simplification of information, warnings, change of layouts (such as menus), positive associations, product placement (making given products more visible or more readily accessible), and framing choices with important social norms and values [43,49,51]. Such interventions work. A meta-analysis has revealed that nudging is effective in guiding consumer choices, including those concerning what we eat [50]. In fact, nudging has been manifested to be more effective in influencing behavior than strict rules or legislation—thus, nudging individuals to eat more vegetarian food works better than forcing them to choose meat-free Mondays [52]. 

Therefore, nudging can help one’s values to align with one’s actions [48]. Importantly, it need not forsake reasoned decision-making altogether. In fact, for nudging to be effective, it needs to be combined with information and deliberation: Individuals, who already accept or are open to a given value, are more susceptible to nudging [43,47]. Interestingly, nudging therefore tends to work precisely in situations of akrasia, where one holds given information and reflected values, but fails to act upon them. Indeed, evidence suggests that it may be one powerful solution to also omnivore’s akrasia, as it can significantly decrease meat-consumption [20,50,51]. In one study, making vegetarian dishes more salient by increasing their visibility on the menu led to a 40 percent increase in popularity. This appeared to have habit-forming, long-lasting benefits, since vegetarian options retained much of their popularity even after nudging had ended [20]. Similar results have been obtained when it comes to climate-friendly eating in general [53] (There are limitations to nudging, one of which is contextuality. Individuals respond differently to “plant-based nudging” depending, for instance, whether they are frequent or infrequent plant-based eaters [51]). Therefore, in light of empirical studies, nudging is one effective method of decreasing omnivore’s akrasia. When individuals are both given information concerning nonhuman beings and their treatment, and nudged so that their unconscious mental states steer them toward choices that align with the information, akrasia becomes less likely. By focusing on the non-rational parts of our minds and making them work for rather than against reason, akrasia loses much of its basis. 

Yet, although nudging is a highly important tool if plant-based eating is to become more widespread, it comes with potential practical and philosophical problems. One concerns the contingency of nudging in a world heavily sculpted by marketing forces. If we rest on manipulations of the unconscious elements in order to put animal ethics into practice, how are we to contest the powers of manipulation that large industries and marketing have? Here, the David of animal ethics may end up battling the Goliath of animal industries without finding his stone. 

Another problem concerns the downplaying of moral agency and its impact on our understanding of humanity. Do we wish to approach human beings in primarily mechanistic terms, as easily manipulated creatures, whose moral choices will inevitably take place outside of awareness, or do we wish to highlight also our moral ability, inclusive of virtues that allow us to intentionally align our choices with our values? Here, the distinction between the descriptive and prescriptive levels is again important. Descriptively, we may indeed exist predominantly in the clutches of “the system 1”, but this need not be the case prescriptively. The cultivation argument gains its impetus from here: Human beings are constantly evolving, and should seek to advance and strengthen also their conscious, intentional moral agency. We need nudging, but we also need the cultivation of moral ability—also in the context of animal ethics. In fact, such cultivation may be the very stone needed by the metaphoric David. 

## 7. Conclusions

Both research on the meat paradox and philosophy on omnivore’s akrasia deserve attention in an era characterized by stark contradictions in how animals are valued and treated. Whereas research on the meat paradox predominantly highlights the role of descriptive mental states, philosophical studies on akrasia, applied in this article to omnivore’s akrasia, mostly emphasize prescriptive, value-laden mental states. Although noting the relevance of descriptive factors is pivotal, this article suggests that paying focus also on the cultivation of moral agency, inclusive of the virtues of self-control, temperance, good will, generosity, and joy in knowledge, is essential. Thus, if animal ethics is to become less theory and more practice, emphasis on descriptive, largely pre-conscious states and methods such as nudging needs to be accompanied by enhancement of moral ability. The specifics of what the key elements of such ability in our dealings with nonhuman creatures comprise of require much further exploration. Indeed, this line of interdisciplinary research forms one fresh and important direction for contemporary animal ethics to participate in.
